# Long‐term effectiveness of a novel intra‐oral electro‐stimulator for the treatment of dry mouth in patients with Sjogren's syndrome: A randomised sham‐controlled feasibility study (LEONIDAS‐1)

**DOI:** 10.1111/jop.13452

**Published:** 2023-05-23

**Authors:** Stefano Fedele, Arwa Al‐Hamad, Valeria Mercadante, Stephen Porter, David Isenberg, Ana Poveda‐Gallego, Sarah T. Brown

**Affiliations:** ^1^ UCL Eastman Dental Institute, University College London London UK; ^2^ NIHR UCLH Biomedical Research Centre London UK; ^3^ King Abdulaziz Medical City National Guard Health Affairs Riyadh Saudi Arabia; ^4^ College of Dentistry King Saud Bin Abdulaziz University for Health Sciences Riyadh Saudi Arabia; ^5^ Centre for Rheumatology, Division of Medicine University College London London UK; ^6^ Oral Medicine Birmingham Dental Hospital and School of Dentistry Birmingham UK; ^7^ Clinical Trials Research Unit, Leeds Institute of Clinical Trials Research University of Leeds Leeds UK

**Keywords:** salivary glands, whole unstimulated salivary flow rate

## Abstract

**Background:**

Effective treatments for dry mouth of Sjogren's syndrome are limited and hampered by adverse effects. The aim of LEONIDAS‐1 was to explore the feasibility of salivary electrostimulation in individuals with primary Sjogren's syndrome, as well as parameters required to inform the design of a future phase III trial.

**Methods:**

Multicentre, parallel‐group, double‐blind, randomised sham‐controlled trial in two UK centres. Participants were randomised (1:1, computer‐generated) to active or sham electrostimulation. The feasibility outcomes included screening/eligibility ratio, consent, and recruitment and drop‐out rates. Preliminary efficacy outcome included dry mouth visual analogue scale, Xerostomia Inventory, the EULAR Sjögren's syndrome patient reported index‐Q1, and unstimulated sialometry.

**Results:**

Forty‐two individuals were screened, of whom 30 (71.4%) met the eligibility criteria. All eligible individuals consented to recruitment. Out of the 30 randomised participants (active *n* = 15, sham *n* = 15), 4 dropped out and 26 (13 vs. 13) completed all study visits as per protocol. Recruitment rate was 2.73 participants/month. At 6‐month post‐randomisation the difference in mean reduction in visual analogue scale, xerostomia inventory and EULAR Sjögren's syndrome patient reported index‐Q1 scores between groups were 0.36 (95% CI: −0.84, 1.56), 3.31 (0.43, 6.18), and 0.23 (−1.17, 1.63), respectively; unstimulated salivary flow increased by a mean of 0.98 mL/15 min, all in favour of the active group. No adverse events were reported.

**Conclusion:**

LEONIDAS‐1 results support progression to a phase III definitive randomised controlled trial of salivary electrostimulation in individuals with Sjogren's syndrome. Xerostomia inventory could be considered the primary patient‐centred outcome measure and the corresponding observed treatment effect could inform the sample size of a future trial.

## 1 INTRODUCTION

Salivary gland hypofunction (dry mouth) is a cardinal feature of Sjogren's syndrome (SS).^1^ It is reported by the vast majority of SS patients (>90%) and the first manifestation of the disease in more than half of cases (up to 54%),^2^ leading to a significant reduction in oral health‐related and general quality of life.^3^, ^4^, ^5^, ^6^ The treatment of salivary hypofunction of SS is a challenge for clinicians (summary of current evidence in Data S1).^7^, ^8^, ^9^, ^10^, ^11^

Neuro‐electrostimulation of the parasympathetic and sympathetic autonomic nerve pathways regulating salivary secretion is considered an attractive non‐pharmacological intervention^12^, ^13^ that may potentially tackle the unmet need of an adverse effect‐free treatment of dry mouth of SS. However its effectiveness in the SS population is unclear. A meta‐analysis conducted in 2019 assessed two double‐blind randomised sham‐controlled studies^14^, ^15^ recruiting a total of 100 SS participants and reported an increase in unstimulated salivary flow associated with the use of a first‐generation intra‐oral electro‐stimulating device, although the quality of evidence was low.^9^ A subsequent multicentre study using a second‐generation custom‐made electro‐stimulating device (the same device as in LEONIDAS‐1) also reported a reduction in dry mouth symptoms and increased salivation^16^, ^17^; however, the quality of evidence was again considered low.^9^ Hence, there remains currently little evidence regarding the clinical effectiveness of salivary neuro‐electrostimulation in individuals with SS. Further robust research in the form of a well‐designed long‐term multi‐centre randomised controlled trial (RCT) is needed to test the hypothesis that the application of an intra‐oral electro‐stimulating device can reduce dry mouth symptoms, increase salivary function, and improve quality of life.

The aims of LEONIDAS‐1 (*Long‐term effectiveness of a novel intra‐oral electro‐stimulator for the treatment of dry mouth in patients with Sjogren's Syndrome: a feasibility study*) were to explore the feasibility and acceptability of salivary electrostimulation using a removable second‐generation custom‐made intra‐oral electro‐stimulating device in individuals with primary SS, as well as a number of study parameters needed to ensure the most appropriate design of a future phase III study including estimates of recruitment rate, compliance with the study intervention, and attrition. A further aim was to obtain preliminary data to quantify the ‘effect’ of the intra‐oral electro‐stimulating device upon dry mouth symptoms and salivary gland function to inform the sample size for a phase III study. The study is reported according to the CONSORT guidelines, extension to randomised pilot and feasibility trials.^18^

## 2 METHODS

### 2.1 Study design

LEONIDAS‐1 was a multicentre, prospective, parallel group, double blind and randomised sham‐controlled trial, on patients with primary SS. Participants were randomised to receive 6 months of therapy with a removable active intra‐oral neuro‐electro‐stimulating device or a sham device (releasing tactile but not electrical stimulation). Participants were followed‐up at 1, 3 and 6 months, post‐randomisation. The subgroup of participants recruited at the lead study site were invited to attend a further standard of care hospital review at 12 months post‐randomisation (open‐label phase—6 months after suspending the study intervention).

LEONIDAS‐1 received favourable opinion by the NRES Committee Yorkshire & The Humber‐Sheffield (Reference: 11/YH/0423) and was sponsored by the Joint UCL/UCLH/Royal Free Biomedical Research Unit. Trial registration number was ISRCTN58887461. Participants provided written informed consent before trial‐related activities commenced.

### 2.2 Study Setting

The study was undertaken in two clinical centres in the UK (outpatient clinics of the Oral Medicine and Rheumatology units of University College London Hospital [UCLH] and Birmingham Community Healthcare NHS Trust) by clinicians with expertise in the management of dry mouth of SS who regularly receive referrals from Rheumatology units and general medical and dental practitioners.

### 2.3 Participants

The full list of study inclusion and exclusion criteria is presented in Table 1.^19^

**TABLE 1:** Study inclusion and exclusion criteria.

Inclusion criteria
≥18 years of age. Symptoms of xerostomia (dry mouth) due to primary SS syndrome diagnosed as per 2001 EU‐USA classification criteria.^19^ A minimum degree of dry mouth symptoms of 50 mm (≥50 mm) on a 100 mm VAS scale (0 = no dryness; 100 = maximum dryness). No systemic sialogogue therapy (e.g., pilocarpine) for the duration of the study. Not be pregnant or trying to have children for the length of their participation. Female participants of child bearing potential would need to take measures to avoid pregnancy. To understand and consent in writing to the procedure. To agree to undergo all the examinations and clinical evaluations of the study. To have evidence of residual salivary gland function, by demonstrating an increase in salivary flow on appropriate stimulation (e.g., citric acid stimulation or chewing paraffin wax). To have unstimulated whole salivary flow higher than 0 mL/15 min (unstimulated salivary flow as measured via sialometry for 15 min).
Exclusion criteria
1 Severe systemic disease (on the basis of the classification of the American Society of Anesthesiology: ASAIII and ASA IV). 2 Being pregnant or trying to have children. 3 Known allergy to materials similar to those used in the investigational product. 4 To wear other active implants such as cardiac pacemaker or defibrillator, or hearing aids. 5 Complete lower edentulous status (i.e., no lower teeth). 6 To have oral anatomical or disease‐related characteristics that preclude the insertion of the device (e.g., mandibular torus and severe trismus). 1 To be unable or unwilling to cooperate with study procedures. 2 To have evidence of no residual salivary gland function (via citric acid stimulation or chewing paraffin wax test). 7 To have an unstimulated whole salivary flow = 0 mL/15 min (absence of unstimulated salivary flow as measured via sialometry for 15 min).

### 2.4 Intervention and Comparator

#### 2.4.1 Active device

The device tested in LEONIDAS‐1 was a CE‐marked second‐generation removable custom‐made intra‐oral salivary neuro‐electrostimulator manufactured by Saliwell Ltd (Israel).

Further details of the electro‐stimulating device are provided in the Data S1. Participants were asked to use the device for a maximum of 5 min per hour, for as many times as they wanted during the day.

#### 2.4.2 Sham device

The sham device's manufacturing and delivery process, as well as the appearance, was identical to the active device. However, the sham device did not deliver electric pulses.

### 2.5 Study procedures

#### 2.5.1 Screening Visit

Potentially eligible participants were identified among patients with primary SS, provided with verbal and written information about the study, and invited to attend a screening visit for eligibility confirmation including the collection of unstimulated and stimulated sialometry and xerostomia visual analogue scale (VAS) score (details in the Data S1). Participants meeting the eligibility criteria had an impression of the lower dentition taken at the end of the screening visit, which was then sent to the device manufacturer. Demographic information was collected.

#### 2.5.2 Randomisation

Participants were randomised in a 1:1 allocation ratio to active neuro‐electro‐stimulating device or a sham device. The randomisation sequence was computer‐generated using stratified random permuted blocks (block size 4), with stratification by centre. Participants were randomised via an independent and central service provided at the Leeds CTRU, to ensure allocation concealment. Further information is provided in the Data S1.

#### 2.5.3 Blinding

The study was double‐blinded, with study investigators and participants unaware of the treatment group allocation.

#### 2.5.4 Baseline visit

During the baseline visit, the allocated device was fitted and adjusted as needed so to ensure comfort and direct contact of the electrodes to the mandibular mucosa. The participants were instructed and trained regarding the modality of use of the device during the 6 months of the study. Written manufacturers' instructions were also provided.

Assessments conducted included dry mouth symptoms and salivary flow (see outcome measures below).

#### 2.5.5 Follow‐up assessments at months 1, 3 and 6 post‐randomisation

The assessments were repeated at month 1, 3 and 6 post‐randomisation, with the visits running preferentially at the same time of the day as the baseline study visit so to minimise fluctuations related to the circadian rhythm of salivary secretion. Participants were also asked to complete a diary of the frequency of application of the device per day. Adverse events (harms) related to the intervention were monitored and recorded at study visits and any point in time as reported by the participants.

Saliva samples were collected during study visits and stored at −80°C for future mechanistic studies.

#### 2.5.6 Study outcome measures

The feasibility outcome measures included screening/eligibility ratio, willingness of eligible patients to participate and be randomised into a double‐blind sham‐controlled trial (consent rate among eligible individuals), recruitment rate and acceptability of the intervention including withdrawal rate and frequency of use of the study device (number of applications). Preliminary efficacy primary outcome measures included dry mouth symptoms, evaluated through a 0–100 mm VAS^20^ and Xerostomia Inventory (XI),^21^ and main *sicca* symptoms of SS, measured using the EULAR Sjögren's syndrome patient reported index (ESSPRI‐Q1).^22^ Preliminary efficacy secondary outcomes included salivary flow, measured using unstimulated sialometry.^23^

#### 2.5.7 Sample size

A sample size of 30 participants was pre‐specified, based on the recommendations for assessing feasibility outcomes and obtain preliminary estimates of response and variability.^24^ The target sample size was considered realistic based on the existing databases of SS patients at the study sites, as well as a contingency plan of contacting further potential participants on the UK Sjogren's syndrome registry.^25^

### 2.6 Statistical analysis

The feasibility outcomes measures were summarised descriptively stratified by recruiting site and arm post‐randomisation. Summaries included the screening/eligibility ratio, consent rate, recruitment rate per month and acceptability of the intervention (withdrawals and device usage).

Analysis of the efficacy outcomes was conducted on the complete case population, by randomised treatment group allocation. As this was a feasibility study, no formal tests were conducted, the focus being on estimation of the treatment effect and corresponding precision.

Univariable linear regression was used to estimate the treatment effect on the change in score from baseline at 6 months post‐randomisation. Summary statistics for the reduction in scores at 6 months post‐baseline for the preliminary efficacy outcome measures, change in score for salivary flow and raw scores at each time point are presented by treatment group.

Mean profiles over time (baseline, end of months 1, 3 and 6) are presented for the preliminary primary and secondary efficacy outcomes. Univariable linear regression was used to estimate the treatment effect on the reduction (or change as appropriate) in score from baseline to 6 months post‐randomisation. Corresponding difference in adjusted mean reduction (or change as appropriate) from baseline to 6 months are presented by treatment group.

A pre‐planned exploratory analysis of VAS scores, XI and ESSPRI‐Q1 scores collected during the open‐label phase at 12 months post‐randomisation (6 months after suspending the study intervention) for the subgroup of participants at the lead recruiting site are reported as the difference in adjusted mean reduction from baseline and corresponding 95% CIs.

### 2.7 Role of funding sources

Detailed in the Data S1.

## 3 RESULTS

### 3.1 Participant flow

**FIGURE 1:**
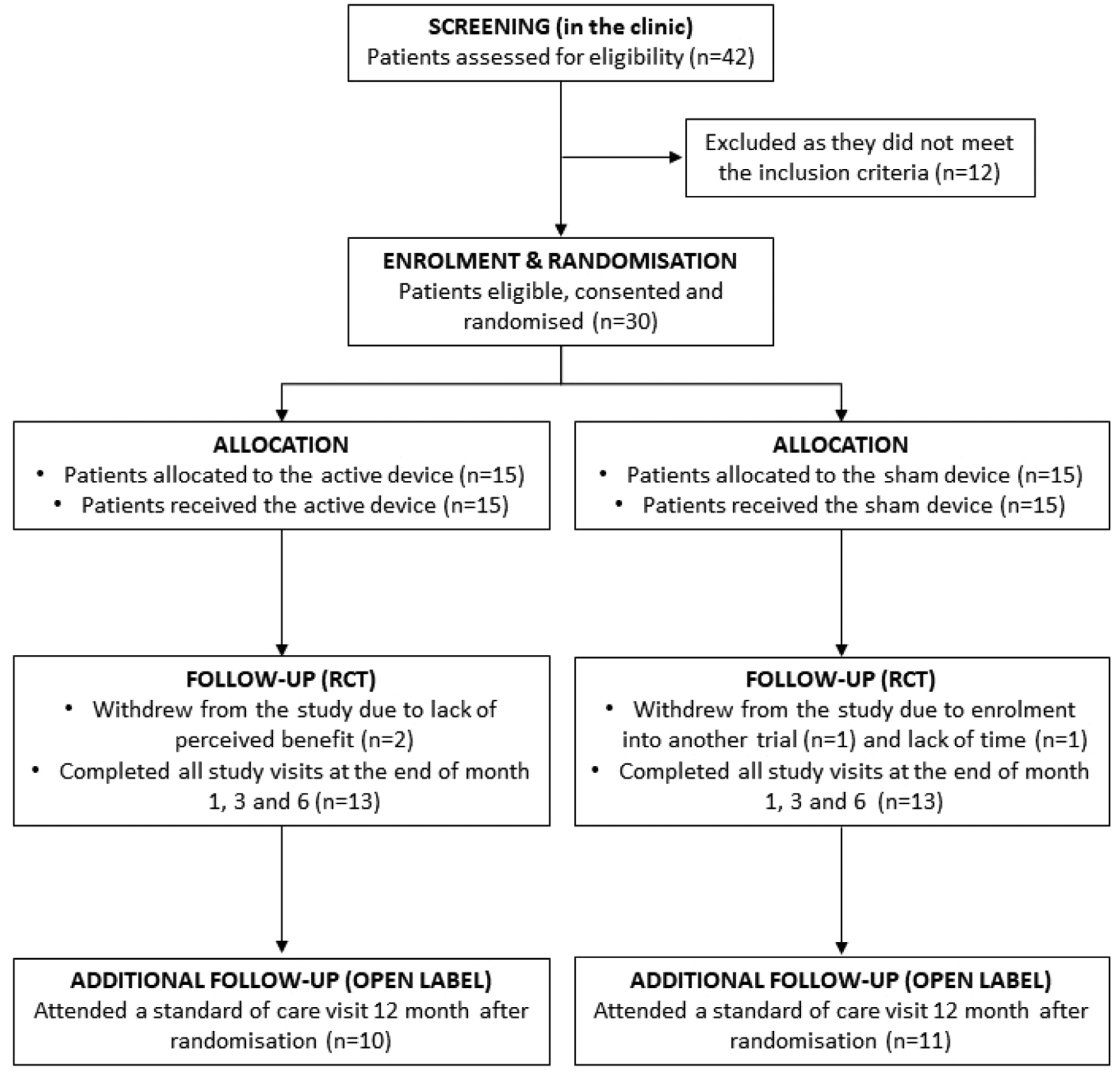
CONSORT flow chart.

Between March 2012 and January 2013 potential participants were identified and approached during standard of care clinics at the two study sites. There was no requirement to use the UK Sjogren's Syndrome Registry due to achieving the target sample size. Forty‐two patients were screened, of whom 12 (28.6%) were excluded due to not meeting the eligibility criteria. The remaining 30 patients (71.4%) were eligible, and all gave written consent to participate in the trial, 25 at UCLH and 5 at Birmingham Community Healthcare NHS Trust. Fifteen (50%) were allocated to receive the active device, and 15 to receive the sham device (50%). The first participant was randomised on 24 April 2012, and the final participant on 22 January 2013. The participant flow is reported in the CONSORT flow diagram (Figure 1). Twenty‐six participants (86.6%) attended all the study visits as per protocol, 13 in each study arm. Four participants (two in the active device and two in the sham group) recruited at UCLH withdrew from all aspects of the study. There were no protocol deviations or violations reported. The baseline demographic and clinical characteristics for each group are presented in Table 2. The mean age of the participants recruited was 61.4 years (range: 44–79) and almost all were female (29/30, 96.7%). Most participants were of white ethnicity (27/30, 90%). Median (IQR) duration of SS at the point of recruitment was 6 years (2.8, 11.0) and baseline unstimulated salivary flow [mean (SD)] was 0.68 mL/15 min (0.74). The mean (SD) baseline scores for XI, dry mouth VAS and ESSPRI Q1 were 46.2 (6.38), 70.5 (1.61) and 8.3 (1.2), respectively. There were no notable differences between the study groups at baseline, except SS duration, which was longer in the sham group.

**TABLE 2:** Baseline demographics and clinical characteristics.

	Active device (*n* = 15)	Sham device (*n* = 15)	All participants (*n* = 30)
Age (mean, range)	61.4 (44, 79)	61.8 (31, 82)	61.6 (31, 82)
Female [*n*/*n* (%)]	15 (100%)	14 (93.3%)	29/30 (96.7%)
Ethnicity [*n*/*n* (%)]	White 14 (93.3%)	White 13 (86.7%)	White 27 (90%)
	Asian 1 (6.7%)	Asian 0 (0.0%)	Asian 1 (3.3%)
	Black 0 (0.0%)	Black 1 (6.7%)	Black 1 (3.3%)
	Other 0 (0.0%)	Other 1 (6.7%)	Other 1 (3.3%)
SS duration (years) [median (IQR)]	3.0 (2.0, 14.0)	7.0 (4.0, 10.0)	6.0 (2.8, 11.0)
Unstimulated salivary flow (mL/15 min) [mean (SD)]	0.68 (0.61)	0.69 (0.88)	0.68 (0.74)
XI score [mean (SD)]	47.3 (6.5)	45.4 (6.1)	46.2 (6.38)
Dry mouth 0–100 mm VAS [mean (SD)]	70.4 (1.8)	70.5 (1.5)	70.5 (1.61)
ESSPRI‐Q1^a^ [mean (SD)]	8.4 (1.4)	8.3 (1.1)	8.3 (1.2)

^a^ ESSPRI‐Q1 data were collected only for participants recruited at UCLH.

### 3.2 Feasibility outcomes

Overall screening/eligibility ratio was 42:30 (71.4% of the screened individuals were eligible across the two study sites). A ratio of 5:5 and 37:25 at Birmingham Community Healthcare NHS Trust and UCLH, respectively, was observed. All eligible patients consented to participation, therefore the ‘acceptability or willingness’ of eligible individuals to participate and be randomised into the study (consent rate) was 100%. The overall mean recruitment rate across both sites was 2.73 participants per month, corresponding to 30 participants recruited over 11 months. UCLH recruited 25 participants over 11 months (mean rate of 2.27/month) and Birmingham Community Healthcare NHS Trust recruited 5 participants over 5 months (mean rate of 1/month). A total of four participants (13.3%) withdrew from the study due to lack of compliance/no benefit perceived (*n* = 2, active arm), travelling (*n* = 1, sham arm), joined another trial (*n* = 1, sham arm). Among those participants who completed the trial, median use of the device was three times/day (range 0–11) in the active group and three times/day (range 0–10) in the sham group.

### 3.3 Preliminary efficacy outcomes

**FIGURE 2:**
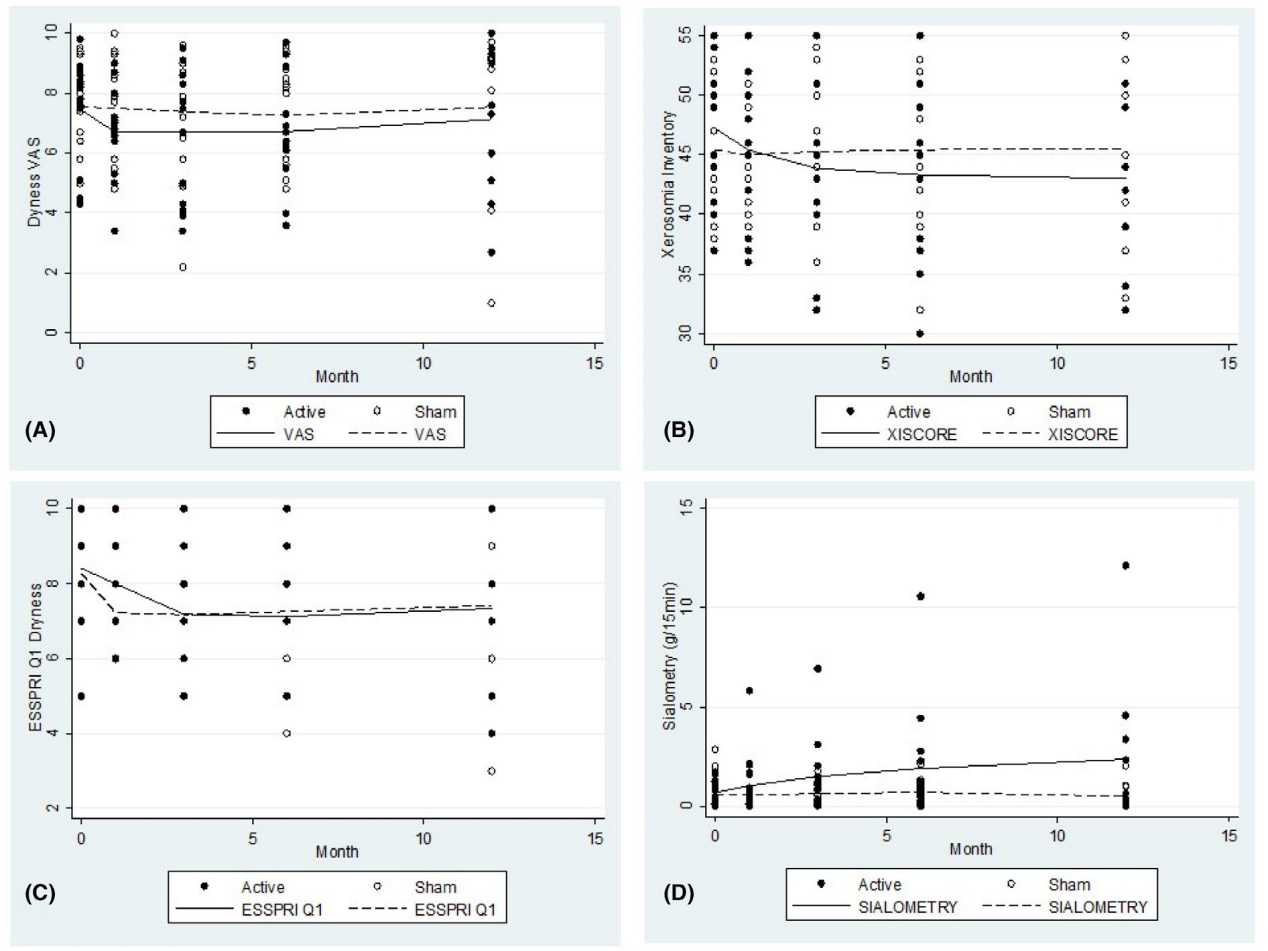
Adjusted mean VAS score (A), XI (B), ESSPRI‐Q1 (C) and salivary flow (D) over 12 months by treatment group.

A total of 26 participants (13 per treatment group) completed all study visits and therefore included in the preliminary efficacy primary and secondary outcome analyses. Data relevant to 21 participants at UCLH who attended the open‐label visit at 12 months post‐randomisation were included in the exploratory analyses. The mean profiles for each of the outcomes over time are presented in Figure 2A–C. Summary statistics are presented in Tables 3 and 4.

**TABLE 3:** Scores over time from baseline to 6 and 12 months by treatment group.

		Baseline	Month 1	Month 3	Month 6	Month 12^a^
VAS	Active	7.4 (1.8)	6.8 (1.5)	6.3 (2.3)	6.9 (1.9)	7.1 (2.5)
	Sham	7.5 (1.5)	7.7 (1.6)	7.3 (2.0)	7.3 (1.7)	7.5 (2.8)
XI	Active	47.3 (6.5)	45.5 (6.6)	43.6 (7.1)	43.5 (7.7)	43.1 (6.6)
	Sham	45.4 (6.1)	44.9 (5.4)	45.5 (6.6)	45.3 (6.8)	45.5 (7.7)
ESSPRI‐Q1	Active	8.4 (1.4)	7.6 (1.3)	7.1 (1.5)	7.2 (1.6)	7.3 (1.9)
	Sham	8.3 (1.1)	7.2 (1.2)	7.2 (1.4)	7.3 (1.7)	7.4 (2.3)
Salivary flow (mL/15 min)	Active	0.7 (0.6)	1.1 (1.5)	1.4 (1.9)	1.9 (2.9)	2.4 (3.8)
	Sham	0.7 (0.9)	0.6 (0.6)	0.6 (0.5)	0.8 (0.6)	0.5 (0.6)

^a^ Data at 12 months were collected only for participants recruited at UCLH.

**TABLE 4:** Reduction (or change) in score at 6 months post‐baseline and overall difference.

		Reduction at month 6 post‐baseline^a^	Difference (95% CI)
VAS	Active	0.7 (1.7)	0.36 (−0.84, 1.56)
	Sham	0.4 (1.3)	
XI	Active	3.5 (4.1)	3.31 (0.43, 6.18)
	Sham	0.2 (3.0)	
ESSPRI‐Q1	Active	1.3 (2.1)	0.23 (−1.17, 1.63)
	Sham	1.1 (1.2)	

		Change at month 6 post‐baseline^b^	Difference
Salivary flow (mL/15 min)	Active	1.2 (2.5)	0.98 (−0.48, 2.45)
	Sham	0.2 (0.6)	

^a^ Reduction defined as baseline score—month 6 score.

^b^ Change defined as month 6 score—baseline score.

#### 3.3.1 Primary outcomes

VAS scores: An initial reduction in mean VAS score in the active group was observed, however the mean VAS score in the sham group remained fairly constant over time. By 6 months post‐randomisation, the gap between treatment groups had almost closed (Figure 2). The difference in the mean reduction in VAS scores at 6 months post‐randomisation was 0.36 units (95% CI: −0.84, 1.56), with a greater reduction in the active group compared to the sham group.

XI scores: A greater reduction in XI score was observed in the active group than in the sham group (Figure 2B). At 6 months, the XI score reduced by a mean of 3.31 units more in the active group than in the sham group (95% CI 0.43, 6.18).

ESSPRI‐Q1: Little difference in *sicca* symptoms was observed between treatment groups; the corresponding difference in the mean reduction in dryness (ESSPRI‐Q1) at 6 months was 0.23 (95% CI: −1.17, 1.63). (Figure 2C).

#### 3.3.2 Secondary outcome

A greater increase in salivary flow was observed in the active group compared to the sham group (Figure 2D). At 6 months post‐randomisation, salivary flow increased by a mean of 0.98 mL/15 min more in the active group than in the sham group (95% CI −0.48, 2.45).

### 3.4 Pre‐planned exploratory outcomes

The mean scores for each of the exploratory outcomes at 12 months post‐randomisation (open‐label phase) are presented in Figure 2C and Table 3.

VAS scores: the gap in VAS scores between treatment groups reduced by 12 months, with a corresponding difference in the mean reduction of 0.3 units (95% CI: −2.1, 2.8) (Figure 2).

XI scores: a greater reduction in the mean XI score at 12 months was observed in the active compared to the sham group, with a corresponding difference between treatment groups of 4.2 (95% CI −0.04, 8.5) units (Figure 2B), suggesting that the reduction in XI score observed at 6 months was sustained at 12 months post‐randomisation.

ESSPRI‐Q1 score: there was little difference observed in the reduction in dryness between treatment groups at 12 months, with a corresponding difference in ESSPRI‐Q1 score of 0.2 (95% CI: −1.8, 2.2) (Figure 2C).

Salivary flow: an increase in salivary flow was observed in the active group at 12 months, with a mean increase of 1.7 g/15 min (95% CI −0.5 to 3.9) relative to the sham group (Figure 2D).

### 3.5 Related adverse event (harms)

No adverse event (harm) related to the study intervention was reported by the participants or attending clinicians.

## 4 DISCUSSION

The screening/eligibility ratio, the consent rate and the recruitment rate observed in LEONIDAS‐1 provided convincing evidence that a future phase III definitive trial is likely to be feasible in NHS secondary care outpatient clinics that routinely care for SS patients. The acceptability of the intervention and the compliance with the study protocol was very good, with all eligible individuals consenting to recruitment, no reported protocol violation/deviations and a very small number of participants (4/30, 13.3%) withdrawing from the study. Of note, only two of these four participants withdrew from the study due to reasons related to the study intervention (no perceived benefit) or protocol (number of study visits), with no participant experiencing notable harm/adverse events. The demographics and clinical characteristics of the study participants at baseline were similar to those reported by other interventional studies in SS patients (e.g., TRACTISS),^26^ therefore suggesting that the LEONIDAS‐1 participants were a fairly representative of the wider SS population enrolled in clinical studies. Nonetheless, the fact that the majority of the participants were recruited at one site (25/30, 83.3%) may represent a potential source of selection bias and a generalisability concern with respect to feasibility and acceptability, suggesting that the future definitive trial would require multiple sites including PIC (patient identification centre) sites. We would also suggest that recruitment rates at both LEONIDAS‐1 sites (2.27/month vs. 1/month) should be considered to inform the expected recruitment rate in the future definitive trial.

A further aim of LEONIDAS‐1 was to obtain data to preliminary assess the effect of the intervention on dry mouth symptoms and salivary gland function, with a view to inform the sample size of the future Phase III definitive trial and identify the outcome measure that is most likely to capture such effect. A preliminary assessment of the efficacy was also important to develop an evidence base to inform investigators and potential funders on the likely beneficial effect of the intervention and the value of a definitive multicentre phase III trial. The primary preliminary efficacy outcomes in LEONIDAS‐1 were all patient‐centred and included two validated measures for dry mouth (0–100 mm VAS and XI) and one validated patient‐reported sicca symptoms index (ESSPRI‐Q1). The secondary preliminary efficacy outcome was the unstimulated salivary flow. The study data showed a greater reduction in the XI score, suggesting that the electro‐stimulating device may effectively reduce dry mouth symptoms in individuals with SS. There was a further reduction in oral dryness as measured by the XI score in the subgroup of participants assessed at 12 months post‐randomisation (6 months after suspending the study intervention) in the pre‐planned exploratory analysis, suggesting a potential long‐term effect of the intervention. This is in keeping with the suggestion that neuro‐electrostimulation may have regenerative potential upon the salivary gland parenchyma.^27^

A minimal, not fully convincing evidence of benefit was identified for the other patient‐centred outcomes or salivary flow, which is expected considering the small scale of this feasibility study not designed to demonstrate efficacy.

## 5 CONCLUSIONS

LEONIDAS‐1 results support progression to a phase III definitive RCT of salivary electrostimulation in individuals with SS, on the basis of the results on feasibility, acceptability, lack of harm and preliminary evidence of efficacy upon dry mouth symptoms. In terms of informing the design of a future definitive trial, LEONIDAS‐1 results suggest that XI could be considered as the primary patient‐centred outcome measure as it was the most sensitive measure capturing the reduction in dry mouth symptoms. No notable major changes in the design, including the inclusion/exclusion criteria, would be considered necessary in the future trial, although it would be reasonable to consider a longer administration of the intervention (e.g., 12 months) to assess the longer term clinical effectiveness. The estimate of the observed treatment effect on the XI score could be used to inform the sample size calculation of a future phase III trial.

## AUTHOR CONTRIBUTIONS

*Study design*: Stefano Fedele and David Isenberg. *Data collection*: Stefano Fedele, Arwa Al‐Hamad, Valeria Mercadante, and Ana Poveda‐Gallego. *Data analysis*: Stefano Fedele, and Sarah T. Brown. *Data interpretation*: Stefano Fedele, Stephen Porter, David Isenberg, and Sarah T. Brown. *Manuscript writing*: Stefano Fedele. *Manuscript Review and Editing*: Arwa Al‐Hamad, Valeria Mercadante, Stephen Porter, David Isenberg, Ana Poveda‐Gallego, and Sarah T Brown. *Study design*: Stephen Porter.

## FUNDING INFORMATION

Funding was provided by Arthritis Research UK (now renamed Versus Arthritis) (Reference 19 709), and further staff support was received from the NIHR Clinical Research Network North Thames and the NIHR UCLH Biomedical Research Centre. The funding source provided peer‐review for the study but had no other role in study design, collection, analysis, interpretation of data or writing of the report. The manufacturer Saliwell Ltd provided the study devices at reduced price for research purpose, and had no role in study design, collection, analysis, interpretation of data or writing of the report.

## CONFLICT OF INTEREST STATEMENT

None of the authors have a conflict of interest to disclose.

### PEER REVIEW

The peer review history for this article is available at https://www.webofscience.com/api/gateway/wos/peer-review/10.1111/jop.13452.

## Supporting information


**Data S1.** Supporting Information.

## Data Availability

The data that support the findings of this study are available from the corresponding author upon reasonable request.
