# Supplementary material for: Long‐term effectiveness of a novel intra‐oral electro‐stimulator for the treatment of dry mouth in patients with Sjogren's syndrome: A randomised sham‐controlled feasibility study (LEONIDAS‐1)
Source: J Oral Pathol Med. 2023 May 23;52(7):619–27. doi: 10.1111/jop.13452 (PMC10946757; doi:10.1111/jop.13452)
Supplement: Supplementary file 1 — Data S1. Supporting Information. [file JOP-52-619-s001.docx]

**SUPPORTING INFORMATION**

1. **Summary of current evidence on treatment of dry mouth of SS**

Historically there has been little robust evidence available to inform and guide clinicians regarding the effectiveness of available interventions.^8,9^ A 2019 systematic review with meta-analysis assessed 36 randomised controlled trials with a pooled total of 3,274 SS patients, and found high quality evidence that pilocarpine can significantly reduce dry mouth symptoms, and moderate evidence that pilocarpine, rituximab and interferon-alpha can increase salivary flow.^9^ However adverse side effects were common (in 34% to 64% of study participants).^9^ Of note, there was no convincing evidence that any of the other reviewed interventions, including the commonly used salivary substitutes and topical sialogogues, are effective in reducing dry mouth symptoms or increasing salivary flow in individuals with SS. ^9^ Real-world observation is in keeping with the above finding, as the majority of SS patients report to rely on drinking frequent sips of water, which only provides minimal transient relief of dry mouth symptoms and does not prevent the many complications associated with lack of saliva.^10,11^

1. **Further details of the electrostimulating device**

*Active device*

The device consisted of a C-shaped mouthguard-like mouthpiece made to fit the lower dental arch, and provided with an associated infrared remote control to activate or deactivate the electrical stimulation. The customised devices were individually manufactured for each participant using moulds of the lower dentition, which were developed from polyvinylsiloxane dental impressions taken by study investigators as per established technique. All devices contained an electronic circuit with a microprocessor, a receiver of remote control signals, a power source of two 3V small coin batteries all embedded and hermetically sealed between two sheets of dental grade acrylic plastic, and had two stimulating electrodes protruding from one side of the plastic sheets so to contact the oral mucosa of the posterior inner aspect of the mandible, nearby the third molar. Fine adjustments were made where necessary on delivery and fitting of the customised devices, with a view to ensure direct contact between the electrodes and the oral mucosa, as well as comfort during use. Adjustments consisted of removing a thin layer of acrylic plastic and/or trimming the electrodes with a dental burr hand-piece and were performed at the discretion of the attending study investigators.

The electrical stimulation was achieved in the active devices by the delivery of low-power, low-voltage, biphasic pulses from the electrodes through the oral mucosa to the nerve pathways of the salivary gland reflex (afferent, efferent or both). The devices were manufactured, sent directly to the study sites, and provided to the study participants during a subsequent study visit (baseline).

The device was switched on and off a remote control, respectively, with a green light on the electronic circuit blinking to show that the device had received the signal from the remote. In case of device failure (device’s light not responding to remote control), a first attempt was made to change the batteries of the remote control. In the event of a permanent failure, a new individual customized device was manufactured.

*Sham device*

The sham device’s manufacturing and delivery process, as well as the appearance, was identical to the active device, including the green light on the electronic circuit blinking when the signal from the remote was received. The device failure procedure was also identical to the one described for the active device.

1. **Screening visit: salivary flow and xerostomia VAS score.**

Unstimulated (resting) whole saliva and paraffin chewing-stimulated whole saliva were collected during the screening visit, and only individuals with a demonstrated increase in salivary flow upon stimulation were recruited in the study. All subjects were instructed to refrain from smoking, eating, drinking or tooth brushing at least 90 minutes prior to saliva collection. Unstimulated whole saliva was collected for 15 minutes using an established spitting technique. During the stimulated whole saliva collection, the subjects chewed a piece of tasteless parafilm at their natural pace. Saliva volumes were determined gravimetrically (assuming 1g = 1mL), using pre-weighted tubes and a precision balance, with saliva flow rates expressed in millilitres per minute (mL/min). The grade of xerostomia at screening was assessed using the xerostomia VAS score (0-100mm).

1. **Randomisation**

At randomisation, each participant was assigned a unique study ID number and received the corresponding device in a pre-packed box; this process was conducted by an independent study coordinator. The active and sham devices were pre-packed in identical boxes, and consecutively numbered for each participant according to the randomisation schedule. The randomisation list was shared with the device manufacturer for the programming of active and sham devices and only disclosed to the study team at the end of the study post statistical analysis.
